# Factors contributing to preventing operating room “never events”: a machine learning analysis

**DOI:** 10.1186/s13037-023-00356-x

**Published:** 2023-03-31

**Authors:** Dana Arad, Ariel Rosenfeld, Racheli Magnezi

**Affiliations:** 1 Department of Management, Health Management Program, Faculty of Sciences, Bar-Ilan University, Ramat Gan, Israel; 2 Department of Information Science, Bar-Ilan University, Ramat Gan, Israel; 3 Patient Safety Division, Ministry of Health, Ramat Gan, Israel

**Keywords:** Never event, Surgery department, Machine learning, Patient safety

## Abstract

**Background:**

A surgical “Never Event” is a preventable error occurring immediately before, during or immediately following surgery. Various factors contribute to the occurrence of major Never Events, but little is known about their quantified risk in relation to a surgery’s characteristics. Our study uses machine learning to reveal and quantify risk factors with the goal of improving patient safety and quality of care.

**Methods:**

We used data from 9,234 observations on safety standards and 101 root-cause analyses from actual, major “Never Events” including wrong site surgery and retained foreign item, and three random forest supervised machine learning models to identify risk factors. Using a standard 10-cross validation technique, we evaluated the models’ metrics, measuring their impact on the occurrence of the two types of Never Events through Gini impurity.

**Results:**

We identified 24 contributing factors in six surgical departments: two had an impact of > 900% in Urology, Orthopedics, and General Surgery; six had an impact of 0–900% in Gynecology, Urology, and Cardiology; and 17 had an impact of < 0%. Combining factors revealed 15–20 pairs with an increased probability in five departments: Gynecology, 875–1900%; Urology, 1900–2600%; Cardiology, 833–1500%; Orthopedics,1825–4225%; and General Surgery, 2720–13,600%. Five factors affected wrong site surgery’s occurrence (-60.96 to 503.92%) and five affected retained foreign body (-74.65 to 151.43%): two nurses (66.26–87.92%), surgery length < 1 h (85.56–122.91%), and surgery length 1–2 h (-60.96 to 85.56%).

**Conclusions:**

Using machine learning, we could quantify the risk factors’ potential impact on wrong site surgeries and retained foreign items in relation to a surgery’s characteristics, suggesting that safety standards should be adjusted to surgery’s characteristics based on risk assessment in each operating room. .

**Trial registration number:**

MOH 032-2019.

**Supplementary Information:**

The online version contains supplementary material available at 10.1186/s13037-023-00356-x.

## Background

Adverse medical events are preventable, unjustifiable errors that can lead to significant morbidity and mortality and increase healthcare expenditures [1]. They are considered to be entirely preventable with the implementation of quality improvement measures [2]. Major Never Events in perioperative care include incorrect surgery sites and foreign items retained in patients following surgery [3, 4].

The human factors approach recognizes that human error is often the result of individual surgeon factors together with work system factors [5], meaning human error is the main contributing factor to Never Events [6]. Human error includes surgeon distraction [7], the surgical team’s lack of situational awareness to possible error, and miscommunication among team members [8]. Additionally, institutional factors and working conditions, including increased workload and clinician pressure, can create a work climate unconducive to meeting the standards required to maintain patient safety [9] and effective teamwork [10].

Currently, two essential international standards aim to reduce Never Event occurrence: (1) the World Health Organization (WHO) Surgical Safety Checklist [11]; and (2) surgical counts of all items used during a surgery [12]. However, incomplete compliance, non-standardized implementation of these standards [13], and other possible unknown factors have meant that the incidence of Never Events has remained unchanged [14]. In Israel, the incidence of retained foreign items during surgery is 3.2 in every 100,000 surgeries [15]. The incidence of wrong site surgeries is unclear but is generally estimated to be 1 in every 100,000 surgeries in Israel.

For this study, we adopted a machine learning approach [16] to identify currently unknown contributors to Never Event occurrence. Previous studies leveraging machine learning methods in health care have demonstrated its advantages in analyzing diverse data types and revealing non-trivial insights compared with traditional methods [17]. To the best of our knowledge, this is the first study using machine learning methods to identify potential contributing factors to the occurrence of Never Events in operating rooms (ORs).

## Methods

### Study design

We utilized a supervised machine learning method known as random forest [18, 19], incorporating the commonly used extra tree classifier [20]. Random Forest is an ensemble learning method that trains multiple “simple” decision tree models and merges them to achieve a more accurate and stable prediction. The use of random forest entails several desired elements needed to properly conduct this study’s analysis. First, random forests are used to rank the importance of features in a natural way, determining their importance by examining to what extent the tree nodes using a feature reduce the impurity (i.e., uncertainty in classification) across all “trees in the forest.” Second, random forests can cope well with imbalanced datasets (as was the case in this study) and avoid overfitting the data. Finally, random forests compared favorably with several other supervised machine learning algorithms we tested using our data, including popular deep neural networks and support vector machines (SVMs). Random forests have been extensively used in the medical field for clinical risk prediction [21] and other applications.

Safety standards used in the operating room (OR) – surgical safety checklists and surgical counts – were divided into safety verifications at three distinct time periods – pre-procedure, sign in, and time out [11] – and addressed incorrect surgery site errors, which we define as type A errors. Surgical counts were divided into three separate counts throughout a surgery to address retained foreign body errors, which we define as type B errors: prior to skin incision; initiation of closure of fascia/cavity; and following skin closure [22]. In addition, we added general features, including the hospital’s name, length of surgery, patient gender and age, surgeon’s specialty, and number of physicians and nurses present during surgery.

### Data collection and annotation

Data were collected from 29 Israeli hospitals and consisted of two types of data entries: observations of 9,234 surgeries performed between January 2018 and February 2019 in which no Never Events occurred during the surgeries observed, and root cause analyses (RCAs) of 101 Never Events that occurred between January 2016 and February 2020 in the examined hospitals.

### Observations

Passive observations by medical students, physicians, nursing students, or registered nurses are routinely performed in ORs under the Israel Ministry of Health’s supervision. Observers for this study underwent an eight-hour long training program that included simulations. In each OR, at least two observers passively observed randomly selected surgeries, recording and annotating the surgery process using a pre-defined set of features. Observations were then transferred to a central database and were run to assess for variability and reliability. Overall, 9,234 observations were conducted. Each observation was translated into a 93-feature-long vector, representing characteristics of the surgery (see Additional file 1). To maintain reliability, entries with greater than 5% discordance among annotators in one OR were discarded (< 1%).

### Root cause analyses (RCAs)

RCAs were performed in response to Never Events occurring between January 2016 and February 2020. We reported 101 Never Events, including 49 of type A and 52 of type B. The obtained RCAs were manually annotated by the authors using the same 93-feature-long representation used to characterize the observations. Unlike the observations, RCAs were performed retrospectively; therefore, a significant portion of the features was missing and could not be obtained. Specifically, up to 40% of all other feature values were missing, a challenge we address later.

### Pre-processing and analysis technique

As some features were non-binary (e.g., patient age, length of surgery), we first discretized them, resulting in 250 binary features. This and subsequent steps were performed using a designated Python 3 program implemented by the authors that uses the standard scikit-learn machine learning package (https://scikit-learn.org/stable).

Examination of the 40% of missing feature values revealed that most were strongly dependent on the Never Event type. Specifically, for type A Never Events, features that were assumed to be more related to Never Events of type B were not investigated and vice versa. For example, for an Never Event in which the wrong hand was operated on, there was no indication as to whether the surgeon scanned the surgical cavity for retained surgical items pre-closure. To mitigate this artifact, we used the iterative data imputation approach [23], predicting the value of each missing value while relying on the present features and available examples. Specifically, using the entire dataset, each missing value was estimated using a standard decision-tree regressor.

In addition, balancing steps were taken to cope with the highly imbalanced dataset. Specifically, with more than 9,000 observations and only 101 Never Events, we adopted a cost-sensitive training approach [24], adjusting our model for prediction mistakes on the minority class (Never Events) by an amount proportional to how under-represented it was (here, approximately 90 times under-represented).

We implemented three random forests models using our data: model 1 to distinguish between observations and Never Events; model 2 for distinguishing between observations and type A Never Events; and model 3 to distinguish between observations and type B Never Events. We used a standard 10-cross validation technique to evaluate each model’s metrics and adopted the standard Gini impurity [25] measure to estimate the importance of features and their combination in our models. Intuitively, Gini impurity captures the “noise” in a set by measuring how often a randomly chosen element from the set would be incorrectly labeled if it were randomly labeled according to the labels’ distribution in the set. We conducted feature importance ranking using the trained random forest models and reported the change in the probability of Never Event occurrence given the entire data set. We considered each feature separately and calculated the probability of Never Event occurrence when that feature assumed the value “True” rather than “False.”

This study was approved by the Ministry of Health’s Ethics Committee (MOH 032-2019).

## Results

The majority of Never Events (62.32%) occurred in six main departments: General Surgery, 19 (18.81%); Gynecology, 17 (16.83%); Orthopedics, 16 (15.84%); Cardiac and Cardiothoracic, 15 (14.85%); Ophthalmology, 8 (7.92%); and Urology, 7 (6.93%) (Table 1). Therefore, our analysis focused on Never Events’ occurrence in these six departments.

**Table 1:** Characteristics of the dataset according to surgical specialty

Observations n = 9234							Never Events n = 101
Phase Specialty	*Pre-procedure (n = 1,539) (missing data on 760 cases)	Sign in (n = 1,504)	Time out (n = 1,498)	First count (n = 1,518)	Second count (n = 1,501)	Third count (n = 1,498)	
Urology	72	156	148	124	118	124	7 (6.93%)
Orthopedics	185	331	324	341	302	326	16 (15.84%)
Ear, nose, and throat	64	105	105	99	102	93	3 (2.97%)
Gynecology	63	143	139	149	153	153	17 (16.83%)
General surgery	313	537	558	576	623	604	19 (18.81%)
Plastic surgery	22	39	37	40	36	42	2 (1.98%)
Vascular surgery	18	45	42	45	42	43	5 (4.95%)
Neurosurgery	7	25	19	22	19	19	5 (4.95%)
Dermatology	7	16	26	21	22	24	2 (1.98%)
Ophthalmology	12	41	34	33	19	18	8 (7.92%)
Maxillofacial	3	12	10	8	10	11	2 (1.98%)
Cardiac and Cardiothoracic	13	54	56	60	55	41	15 (14.85%)

**Table 2:** Characteristics of patients and surgeries in the dataset

Characteristic	Observations	Never Events
**Gender**	Male (n = 388 (49.8%)), Female (n = 391 (50.2%))	Male (n = 46 (45.5%)) Female n = 55 (54.5%)
**Length of surgery**	Up to 1 h: 2124 (23%) 1–2 h: 4340 (47%) 3–4 h: 2031 (22%) Over 4 h: 739 (8%)	Length of surgery: Up to 1 h: 54 (53.5%) 1–2 h: 13 (12.9%) 3–4 h: 17 (16.8%) Over 4 h: 17 (16.8%)

To evaluate our models, we adopted the area under the curve (AUC) measure. This measure is especially suited for imbalanced data, as was the case in this study, as it does not have any bias toward models that perform well on the minority of majority classes at the expense of the other [26]. Our three random forest models each demonstrated good performance, exhibiting an AUC between 0.81 and 0.85. Generally, AUC scores between 0.8 and 0.9 are considered excellent [27]. AUC is interpreted as the probability that our model will rank a randomly chosen positive instance higher than a randomly chosen negative one [28]. As such, our models can be considered relatively strong and accurate, despite their limitations.

### Feature importance

**Fig. 1:**
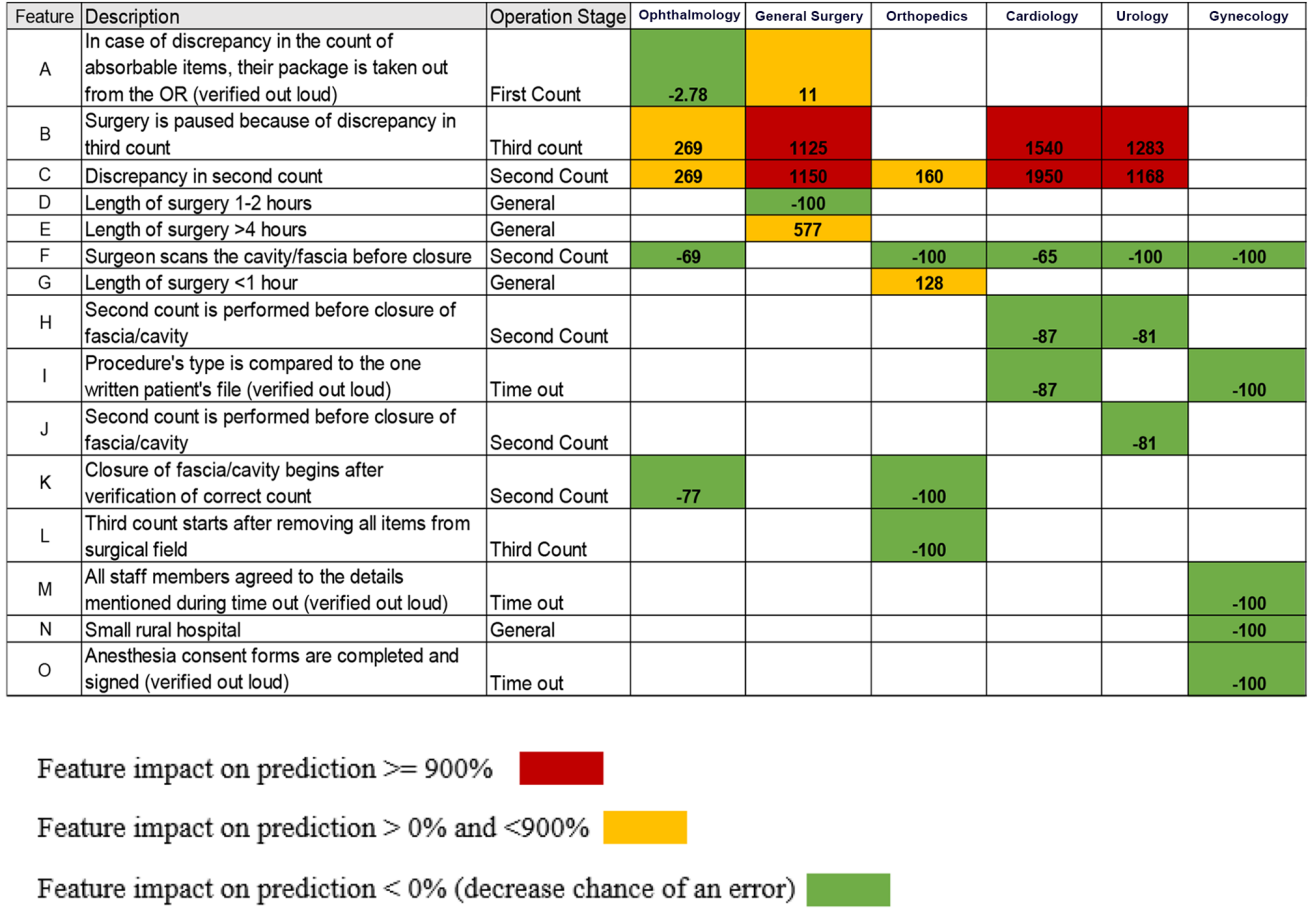
Top 15 contributing features for the six examined departments

Figure 1 shows the most common contributing features to the occurrence of Never Events (of both types combined) in the six departments, along with the associated probability change.

The top 14 contributing features varied significantly across departments, and no single feature set was consistently more informative across all operations for predicting Never Events. For example, feature [C], “Discrepancy in second count,” varied significantly across departments (160% to 1,950%). Feature [B], “Surgery is paused because of discrepancy in third count,” appeared in four of the six departments, and the associated probability change varied dramatically, between 269% and 1,540%. There were 10 features that consistently decreased the chance of a Never Event occurring, including [F] “Surgeon scans the cavity/fascia before closure during the second count,” which affected five out of six departments and was consistent in its probability change, between 65 and 100%. Features [I], [J], [ K], [L], [M], and [N] decreased the chances of Never Events between 2 and 100% in three departments. Three features, [A] “Discrepancy in absorbing materials,” [E] “Surgery time > 4 hours,” and [G] “Surgery time < 1 hour” appeared once across departments, with a medium impact on Never Event occurrence.

Analysis of the results by department shows variation among the contributing features. For example, in Ophthalmology, the probability was consistently − 100% for five features, while in General Surgery, two features that increased the probability of an error varied between 1,168–1,283%: features [B] “Surgery is paused because of discrepancy in third count” and [C] “Discrepancy in second count.” In Orthopedics, those same two features, [B] and [C], increased the probability of error (1,540–1,950%). Three features decreased the probability of error: [F] “Surgeon scans the cavity/fascia before closure”; [H] “Second count is performed before closure of fascia/cavity”; and (I) “Procedure type is compared to the one written in patient’s file,” by -65 to -87%.

### Effects of feature combinations

**Fig. 2:**
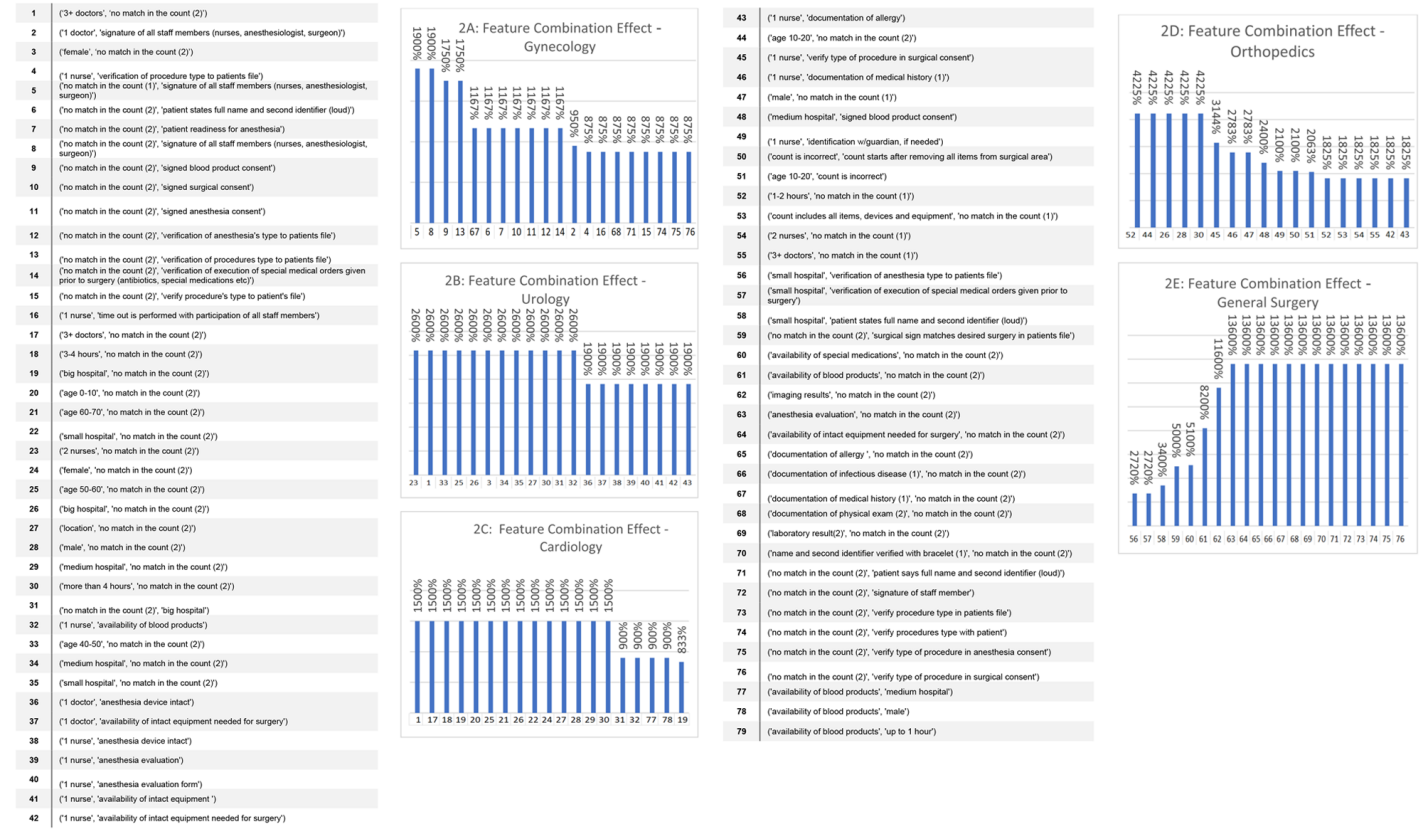
Effect of two features’ combination on prediction by surgical departments

In the following analysis (Fig. 2), we examine the effects of paired features, i.e., features that occur together in the data. It is important to note that when considering feature combinations, their occurrence is expected to be very low, especially in the Never Events class. As such, the estimated effects are likely to be very high, yet their confidence is significantly low.

Interestingly, in General Surgery, there were 14 feature combinations that caused a probability change of 13,600% (Fig. 2A). In comparison, the single feature analysis (Fig. 1) revealed a probability change of 1,287% and 1,168%, surprisingly by two features that were not part of the 14 feature combinations identified here.

In Fig. 2A (Gynecology), the effect of every feature combination is associated with a probability change of 1,000–2,000%. In the single feature analysis (Table 2), the effect of two of the features separately was < 900%, and the rest lagged behind with < 150%. In Urology (Fig. 2B), the results showed there were dozens of pairs with an effect of 1,900–2,500%, while the effect of a single feature had < 1,150% effect on error. In General Surgery (Fig. 2E), the accumulated effect of two features together showed a dozen pairs with an effect of 1,900–4,200%, while the effect of a single feature had a < 1,950% effect on error, and the rest showed even lower percentages.

### Features affecting types a and B

**Fig. 3:**
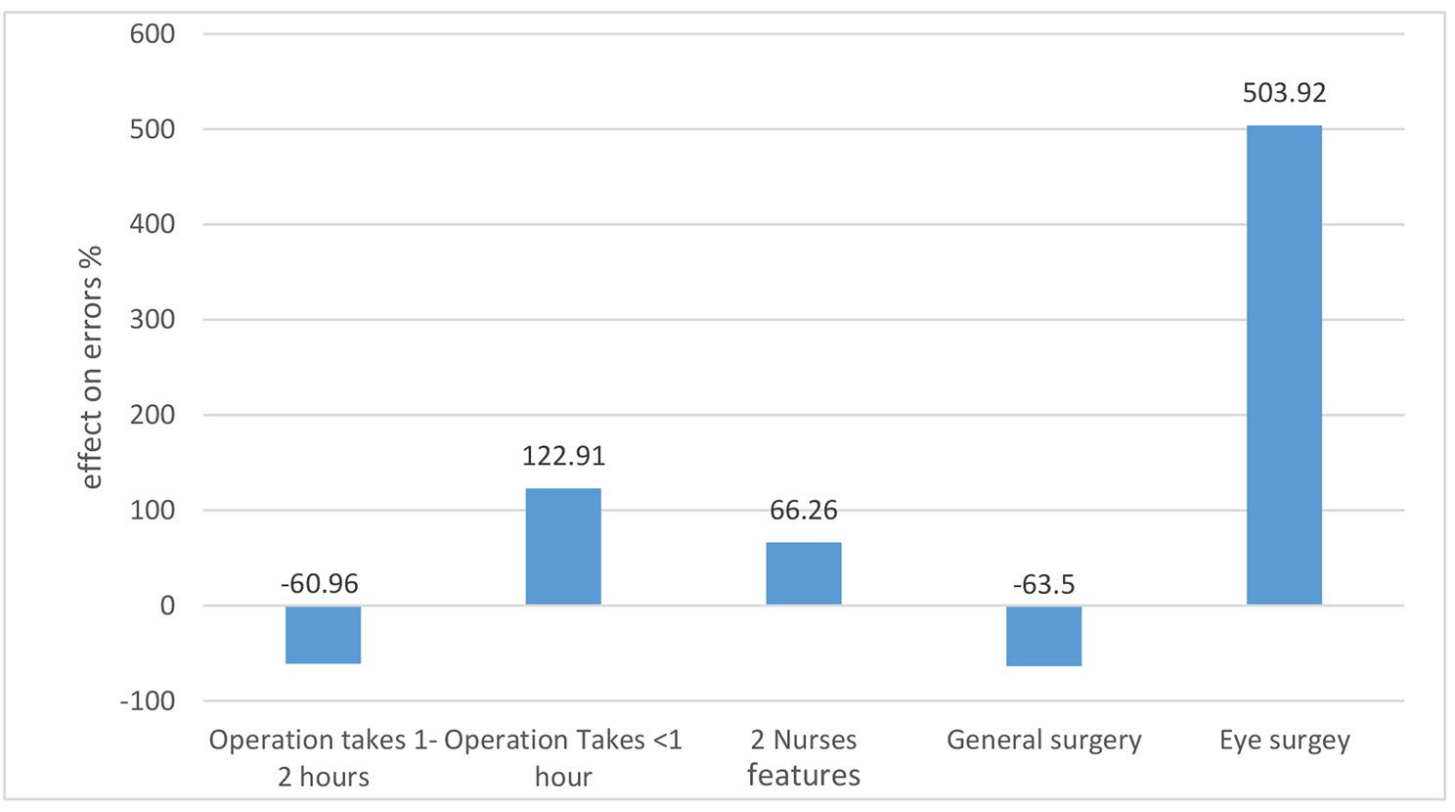
Features affecting the wrong surgery site (type A)

**Fig. 4:**
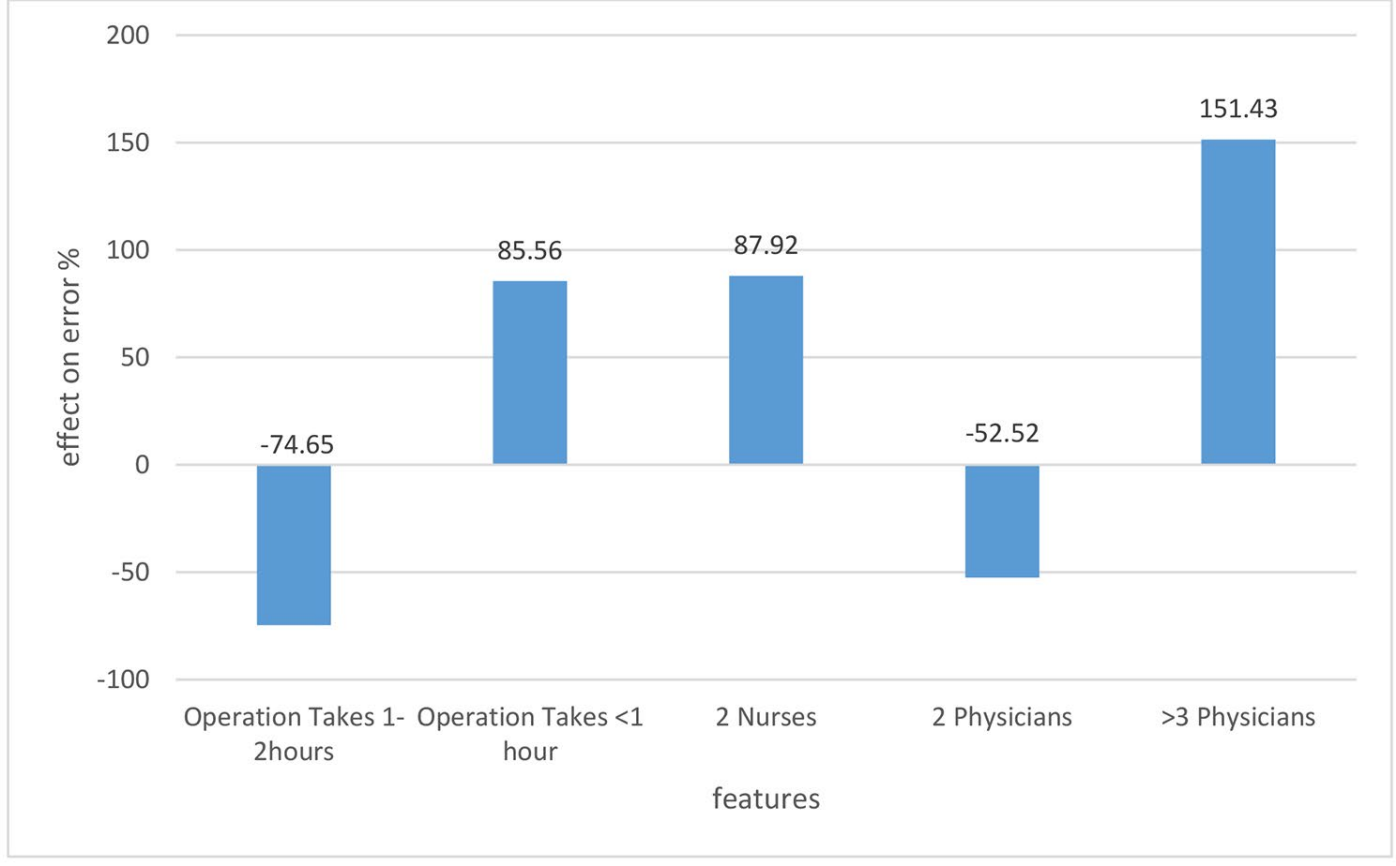
Features affecting retained foreign items during surgery (type B)

Turning to Models 2 and 3, there was an overlap in three of the top five contributing features to type A and B errors (Figs. 3 and 4): (1) the presence of two nurses during the surgery predicted a greater occurrence of type A (66%) and type B (88%); (2) an operation < 1 h had a greater occurrence of type A (122%) and type B (87%); and (3) when the operation lasted between one to two hours, both types A and B were less frequent, decreasing by 60% and 74%, respectively. The surgical department that was most affected regarding the occurrence of type A Never Events was Ophthalmology, with a prevalence of 504%, while General Surgery was associated with a decrease of 63% in type A (Fig. 3). For type B, the two remaining features were staff driven; the feature “more than three physicians” was associated with an increased prevalence of type B (151%), while “two physicians” was associated with a decreased prevalence of Type B, by 52% (Fig. 4).

## Discussion

Surgical errors are a serious public health problem and uncovering their causes is challenging [29]. In this study, we sought to uncover factors that contribute to Never Events by using machine learning methods to identify heretofore unknown contributors, as machine learning can be used to automate searches for patterns not seen when using traditional methods [18, 30].

The checklists used in the OR, mainly the surgical safety checklist and surgical count, aim to implement strict work processes in order to prevent errors. Despite their widespread use, the incidence of Never Events has not significantly decreased [31, 32], probably because their occurrence is related to human error and not to the system errors that have been identified as contributing to Never Events. Such system errors are dependent on staff behavior [31, 33]. For example, in our study, discrepancy in the surgical count was found to be a contributing factor to Never Events, while fascia closure after a correct surgical count or staff’s agreement to time out were protective factors for prevention of Never Events. Another study supported the impact of the human factor in performing safety standards and occurrence of Never Events and classified them into four categories: preconditions for action, unsafe actions, oversight and supervisory factors, and organization influences [6]. Additional studies have described the lack of safety standards implementation in the OR as arising from a lack of communication and note the lack of empirical evidence relating to barriers to their implementation [29, 34]. Our findings revealed the contribution of discrepancies in the surgical count to occurrence of Never Events. Some studies have suggested that surgical counts alone are insufficient; even when declared to be correct, items have been left in patients [35, 36], mostly in the abdomen and pelvis [35, 37]. This may also explain our finding of a higher probability of type B errors in General Surgery and Urology, which involve these regions.

We further analyzed paired contributing factors representing the relative risk in the OR’s complex work environment, when the graded risk increased compared to single feature analysis. For example, in Orthopedics, discrepancy in the count in combination with a surgery length of 1–2 h increased the chances for a Never Event, which can be explained by partial compliance with the safety standards. In shorter surgeries, staff may rush and skip some phases of the checklists [38] and the complex surgical devices used during the surgery challenges the counts [31, 39].

We found that the occurrence of incorrect surgery site increased in Ophthalmology during short surgeries and when two nurses were present. Its occurrence decreased in general surgery. This increased risk could be due to the difficulty of performing a time out because the surgeon’s hands are sterilized and they cannot review charts, or perhaps because doing so is not made a priority [40]. The decreased risk in general surgery could be explained by better implementation of the time out process in that specialty [41, 42].

One of the main factors contributing to the occurrence of Never Events is a lack of communication among members of the surgical team [33], which may explain our finding that the number of staff participating in the surgery had a proportional increasing/decreasing effect on Never Event occurrence – and outcome likely affected by lack of communication.

We recognize that the current study is limited by the quantity, quality, and diversity of the data used. Our samples come from two distinct sources: prospective observations and retrospective investigations of Never Events, the latter consisting of a small number of Never Events compared to the relatively high number of observations analyzed. We believe that these limitations are inherent in the problem studied, as performing prospective analyses of Never Events is virtually impossible due to their infrequency, and the number of Never Events is nominally small. To mitigate some of these concerns, we have used grounded statistical techniques enabling us to train adequate models and estimate feature importance. Nevertheless, given the above, the impact of features should be carefully considered and validated in future studies.

In the future, we plan to further expand our data pool with newly obtained observations and Never Events as they are accumulated. In other work, we will explore the use of transferable learning about Never Events from other countries, which could be used to better inform our model. This approach could prove valuable in mitigating the imbalanced nature of our data, although it could introduce considerable biases due to the variety of data sources.

## Conclusion

In this study, we used machine learning methods to reveal unknown contributing factors to occurrence or prevention of Never Events based on surgery’s characteristics, including type, length, and staff presence. We also quantified the contribution of the use of safety standards to occurrence of Never Events.

Our results suggest that the existing, “one size fits all” safety approach should be adjusted to accommodate the surgery’s characteristics. Specifically, each Operating Room should perform a risk assessment relative to the occurrence of Never Events during a specific surgery and make tailored adjustments in the safety standards or work environment to prevent them.

## Electronic supplementary material

Below is the link to the electronic supplementary material.

Supplementary Material 1

Supplementary Material 2
