# Supplementary material for: Factors contributing to preventing operating room “never events”: a machine learning analysis
Source: Patient Saf Surg. 2023 Mar 31;17:6. doi: 10.1186/s13037-023-00356-x (PMC10067209; doi:10.1186/s13037-023-00356-x)
Supplement: Supplementary file 1 — Supplementary Material 1 [file 13037_2023_356_MOESM1_ESM.docx]

Appendix A Surgical safety standards checklist

**Additional File 1- Structure of Observations (based on MOH regulations)**

- 1. Surgical Checklist

Phase 1. Pre-procedure

| **N/A** | **NO** | **Yes** | **Statement** |
| --- | --- | --- | --- |
|  |  |  | Patient states full name and second identifier |
|  |  |  | Name and second identifier verified |
|  |  |  | Identification w/guardian, if needed |
|  |  |  | Verify procedure type with patient |
|  |  |  | Verify procedure type to patient's file |
|  |  |  | Verify type of procedure in surgical consent |
|  |  |  | Verify type of procedure in anesthesia consent |
|  |  |  | Surgical sign matches the desired surgery |
|  |  |  | Documentation of medical history |
|  |  |  | Documentation of physical exam |
|  |  |  | Documentation of infectious disease |
|  |  |  | Anesthesia evaluation |
|  |  |  | Documentation of allergy |
|  |  |  | Laboratory results |
|  |  |  | Imaging results |
|  |  |  | Availability of blood |
|  |  |  | Availability of medications |
|  |  |  | Availability of equipment |
|  |  |  | Signature |

Phase 2. Sign-in

| **N/A** | **NO** | **Yes** | **Statement** |
| --- | --- | --- | --- |
|  |  |  | Sign-in performed by surgeon, anesthesiologist and nurse |
|  |  |  | Patient states full name and second identifier |
|  |  |  | Name and second identifier verified |
|  |  |  | Verification procedure type to patient's file |
|  |  |  | Verification anesthesia type to patient's file |
|  |  |  | Surgical sign matches the patient's file |
|  |  |  | Readiness for anesthesia |
|  |  |  | Anesthesia device intact |
|  |  |  | Documentation of medical history |
|  |  |  | Documentation of physical exam |
|  |  |  | Documentation of infectious disease |
|  |  |  | Anesthesia evaluation |
|  |  |  | Signed surgical consent |
|  |  |  | Signed anesthesia consent |
|  |  |  | Signed blood product consent |
|  |  |  | Documentation of allergy |
|  |  |  | Laboratory results |
|  |  |  | Imaging results |
|  |  |  | Availability of blood |
|  |  |  | Availability of medications |
|  |  |  | Availability of equipment |
|  |  |  | Execution of medical orders |
|  |  |  | Signature (surgeon, anesthesiologist, nurse) |

Phase 3. Time Out

| **N/A** | **NO** | **Yes** | **Statement** |
| --- | --- | --- | --- |
|  |  |  | Sign-in performed by all staff members |
|  |  |  | Time out is before surgical cut |
|  |  |  | Time out performed with the patient |
|  |  |  | All staff members stop and listen |
|  |  |  | Patient identified by 2 identifiers |
|  |  |  | Procedure compared to patient's file |
|  |  |  | Surgical sign matches the patient's file |
|  |  |  | Signed surgical consent |
|  |  |  | Signed anesthesia consent |
|  |  |  | Time out for each procedure |
|  |  |  | Verbal agreement of all staff members |
|  |  |  | Repeat time out in surgeon’s exchange |
|  |  |  | Signature of all staff members |

- 1. Surgical Counts - Observations By Surgical Phase and Type Of Count:

First Count - Prior to Skin Incision:

| **N/A** | **NO** | **Yes** | **Statement** |
| --- | --- | --- | --- |
|  |  |  | Count performed by scrubbed nurse or two nurses |
|  |  |  | Count is made aloud before the beginning of surgery, while opening the sterile equipment |
|  |  |  | Equipment count is made out loud compared to the list |
|  |  |  | Count of absorbable items is made out loud while separating |
|  |  |  | In case of no matching in absorbable items, it is removed from the OR |
|  |  |  | Documentation of the count on a dedicated form |
|  |  |  | Items are not removed from the OR while counting |
|  |  |  | No match in the count |
|  |  |  | Nurses announce the non-match to surgeon |
|  |  |  | Surgery stops due to non-match |
|  |  |  | Searching the missing item |
|  |  |  | Ordering imaging test for finding the missing item |

Second count- closure of fascia/cavity is initiated:

| **N/A** | **NO** | **Yes** | **Statement** |
| --- | --- | --- | --- |
|  |  |  | Count performed by scrubbed nurse or two nurses |
|  |  |  | Count performed before closure of fascia/cavity |
|  |  |  | Equipment count is made out loud with the participation of all staff members |
|  |  |  | Surgeon announces out loud about intention to closure before closure of fascia/cavity |
|  |  |  | Surgeon reviews the cavity before closure |
|  |  |  | Two nurses perform the count |
|  |  |  | Closure begins after verifying correct count |
|  |  |  | No match in the count |
|  |  |  | Nurses announce the non-match to surgeon |
|  |  |  | Surgery stops due to non-match |
|  |  |  | Searching the missing item |
|  |  |  | Ordering imaging test for finding the missing item |

Third Count - After Skin Closure:

| **N/A** | **NO** | **Yes** | **Statement** |
| --- | --- | --- | --- |
|  |  |  | Count performed by scrubbed nurse or two nurses |
|  |  |  | Count is made after removing items from surgical area |
|  |  |  | Count is made out loud with the participation of all staff members |
|  |  |  | Count includes all items, devices and equipment |
|  |  |  | Count is declared when there are no items left in the surgical field |
|  |  |  | Count is documented in a dedicated form |
|  |  |  | Sterile nurse declares out loud that count is correct |
|  |  |  | Nurses’ names and results of count are documented |
|  |  |  | Surgeon verifies out loud that count is correct |
|  |  |  | **Count does not match** |
|  |  |  | Nurses announce the non-match to surgeon |
|  |  |  | Surgery stops due to non-match |
|  |  |  | Search for the missing item |
|  |  |  | Order imaging test to find the missing item |
