# Supplementary material for: Factors contributing to preventing operating room “never events”: a machine learning analysis
Source: Patient Saf Surg. 2023 Mar 31;17:6. doi: 10.1186/s13037-023-00356-x (PMC10067209; doi:10.1186/s13037-023-00356-x)
Supplement: Supplementary file 2 — Supplementary Material 2 [file 13037_2023_356_MOESM2_ESM.docx]

Dana Arad
Ministry of Health
Bar-Ilan University
danaarad@gmail.com

November 10^th^, 2022

Dear Editor,

I am writing to clarify that this research **was approved by the Ethics Committee of the Israeli Ministry of Health (MOH) on January 15, 2020 (Approval number 1/2020 to trial registration number MOH 032-2019) and was extended on July 23, 2021 until July 23, 2022.**

The approval is based on Public Health Regulations- Clinical experiments on Humans in Israel (2014) and on international Declaration of Helsinki ethical principles for medical research involving human subjects.

The committee has given a waiver from any obligation to obtain informed consent in the study.

On January 15, 2020 the study was approved by the ethics committee (chair, Prof Sitvanit Alon-Shalev) and was extended on July 23, 2021 (chair, Prof Eithan Friedman). The approval was signed by Prof Avi Israeli, the chief scientist officer in the Ministry of Health, on behalf of the Director General.

Thank you for your consideration of this manuscript.

Sincerely,

Dana Arad
